# Awareness and Management of Knee Pain and Osteoarthritis in Saudi Arabia: A Cross-Sectional Analysis

**DOI:** 10.7759/cureus.52736

**Published:** 2024-01-22

**Authors:** Abdulmalik B Albaker, Raghad Mahdi M Al-Awn, Salem M Basalem, Lama Alharbi, Rakan Al Salhi, Khalid M Alkhalifah, Nawaf Alhazmi, Manal Almasary, Yousef M Almohammadi

**Affiliations:** 1 Orthopedics, College of Medicine, Majmaah University, Majmaah, SAU; 2 Medicine and Surgery, King Khalid University Hospital, Abha, SAU; 3 Orthopedic Surgery, King Saud University, Riyadh, SAU; 4 Medicine and Surgery, Al Maarefa University, Riyadh, SAU; 5 Orthopedic Surgery, King Saud Medical City, Riyadh, SAU; 6 Unaizah College of Medicine and Medical Sciences, Qassim University, Unaizah, SAU; 7 College of Medicine, Umm Al-Qura University, Makkah, SAU; 8 College of Medicine, Al Faisal University College of Medicine, Riyadh, SAU

**Keywords:** physical exercise, medical managment, intra articular injection, osteoarthritis, chronic knee pain

## Abstract

Objective: The study sought to assess the level of awareness regarding osteoarthritis and its management.

Methods: This study was cross-sectional, using data from a sample of 389 individuals from the central region of Saudi Arabia. The participants completed an online questionnaire and ensured anonymity.

Results: A total of 389 participants made up the sample for this study, which had a predominance of females (56.6%, n=220), a majority aged <50 years (66.6%, n=259), and most of them (51.7%, n=201) weighing 60-80 kg, substantial proportion lived in the Riyadh region (27.5%, n=107), with more than half (59.4%, n=231) having a university education and working in offices (28.3%, n=110). The majority (73.3%, n=285) of participants were married, and a vast majority (87.9%, n=342) were not smokers. The findings revealed that only 32.9% (n=128) of the participants had good knowledge about osteoarthritis. The study found that stiffness (80.2%, n=312) and swelling (97.9%, n = 381) are the most common signs and symptoms of osteoarthritis; the risk factors for osteoarthritis were genetic factors (79.7%, n=310) and age (91.3%, n=355). The treatment of osteoarthritis identified in the study included exercises such as swimming (85.1%, n=331), physical therapy (86.6%, n=337), and joint replacement surgery (92.0%, n=358). The study established a statistically significant association between age, education level, previous diagnosis of osteoarthritis, family history of osteoarthritis (p = 0.004, 0.001, 0.002, and 0.001, respectively), and level of knowledge about osteoarthritis. However, there was no statistically significant association between gender, marital status, smoking status, previous knee injuries, physical activity level, and the level of knowledge about osteoarthritis (p > 0.05).

Conclusion: Overall, the study revealed that 32.9% (n=128) of the participants had good knowledge about osteoarthritis. Participants aged 50-60 years, those with a university and post-graduate level of education, as well as those who had a previous diagnosis of osteoarthritis and those with a family history of osteoarthritis, had greater and better knowledge and awareness about osteoarthritis. Joint stiffness and swelling were identified, as the most common signs and symptoms of osteoarthritis. The risk factors identified in the study were genetic factors and age, while the treatment options noted by the study were exercise, such as swimming, physical therapy, and joint replacement surgery. The study notes the need for enhanced public awareness of the problems associated with osteoarthritis among the Saudi Arabian population.

## Introduction

Osteoarthritis (OA) is the most common form of arthritis that occurs in individuals. This complex condition can have an impact on various parts of the body, including articular cartilage, bones, ligaments, and the synovium. Its effects encompass degenerative and reparative processes, in addition to causing joint inflammation [1]. OA is not discriminatory in its reach, as it can affect numerous joints in the body, regardless of size or location. However, the knee is the most frequently afflicted joint [2]. There are several factors that contribute to the development of knee OA, which can be divided into non-modifiable or modifiable risk factors [3]. Among the modifiable risk factors, some that are well-known include obesity and being overweight, as well as comorbidities such as diabetes, depression, and cardiovascular disease. Other factors that can be modified include occupational variables, physical activity levels, biomechanical factors, and nutritional intake. The focus of treatment should be on addressing those risk factors that can be modified, as this can potentially lead to a reduction in discomfort [3].

Knee pain and early osteoarthritis, two common musculoskeletal conditions, have become significant health challenges worldwide, including in Saudi Arabia. These conditions adversely affect the quality of life of individuals and burden healthcare systems [4]. With Saudi Arabia experiencing rapid socio-economic and demographic changes, it has become crucial to understand the awareness and management of knee pain and early osteoarthritis among the general population [5]. Thus, this study aims to comprehensively review the existing literature and shed light on the knowledge levels and treatment approaches prevalent in Saudi Arabia.

The prevalence and incidence of knee pain and osteoarthritis are not unique to Saudi Arabia. Several studies have investigated these conditions among the Saudi population. For example, a cross-sectional study conducted in the Aseer Region revealed a significant prevalence of knee pain among Saudi adults aged 17 to 80 years [5]. Another study by Alahmed et al. explored early osteoarthritis and its prevalence among middle-aged women in Aseer Central Hospital, providing valuable insights into regional variations in terms of awareness levels and the knowledge gap [6].

Awareness and knowledge are crucial for the early diagnosis and effective management of knee pain and osteoarthritis. However, limited studies have investigated public awareness of these conditions in Saudi Arabia. Available studies that have assessed awareness levels among the general population indicate a substantial lack of knowledge about the risk factors and symptoms associated with knee pain and osteoarthritis in Saudi Arabia [6,7]. However, a study conducted in the Saudi Arabian region demonstrated excellent awareness levels, which correlated with higher education levels [5]. These findings highlight the need for targeted health education and awareness campaigns in the region.

Effective management of knee pain and early osteoarthritis depends on timely healthcare seeking and appropriate treatment approaches. Research has examined the treatment-seeking behavior and preferences of individuals in Saudi Arabia when faced with these symptoms. Silvian et al. explored the utilization of pharmacological and non-pharmacological approaches in treating knee pain in Saudi Arabia, emphasizing the significance of evidence-based practices in healthcare strategies. According to the authors, pharmacological interventions, such as analgesics and surgical procedures, are available in healthcare settings and aid in managing the disease. Additionally, the authors observed that non-pharmacological interventions, such as lifestyle modifications and physical exercise promotion, have proven beneficial in managing knee pain and osteoarthritis [8]. However, several scholars have noted that self-medication remains the preferred method of disease management among many patients in Saudi Arabia, which may result in ineffective management and delayed treatment [9]. Such practices ultimately affect the quality of life of those affected. Despite the valuable insights from existing research, a knowledge gap persists. Although the prevalence of knee pain and early osteoarthritis continues to increase, there is a lack of comprehensive studies focusing specifically on their awareness and management in Saudi Arabia. The present research aims to bridge this knowledge gap.

## Materials and methods

Study setting

The study was carried out from August 1 to September 30, 2023, with an online questionnaire distributed through social media applications (WhatsApp, Twitter, and Facebook) to invite participants from the central region of Saudi Arabia to participate in the study. 

Target population

Inclusion Criteria

This study's inclusion criteria stipulated the participation of Saudi citizens aged 18 years and older, requiring not only an expressed willingness to engage in the research but also possessing reliable internet access. The selection of the age threshold at 18 years aimed to align with the legal definition of adulthood in the context of Saudi Arabian law.

Exclusion Criteria

The exclusion criteria involved the omission of individuals failing to meet the inclusion standards, particularly non-Saudi citizens. Additionally, individuals below the age of 18 were excluded to ensure a targeted focus on the adult demographic.

Sample Size

The sample size was determined using Epi-Info software, version 7.2.2.6, with a 95% confidence interval and 80% power. Given the absence of a similar study in the central region, we assumed a 50% prevalence of poor knowledge about osteoarthritis. This led to an estimated sample size of 384 participants.

Sampling technique (with inclusion and exclusion criteria): participants were recruited by a convenient sampling technique and requested to sign informed consent. The study’s goals were presented to the participants, and informed consent was obtained. The data was obtained from all participants we reached through an electronic questionnaire that met the following criteria:

Data collection methods 

*Item Generation* 

We followed standard guidelines for the development and validation of questionnaires [10,11]. Comprehensive literature research was conducted, and in-depth discussions took place with colleagues, experts, and individuals possessing relevant knowledge about osteoarthritis. Various questionnaires specific to the field of interest were also employed. Participants played a crucial role by offering valuable input on the items, wording, and overall flow of the questionnaire, mindful of their potential role as future respondents [5,12-14]. 

*Item Formatting* 

In the next phase, the set of questions underwent meticulous organization and revision to eliminate any potential ambiguity, technical jargon, or bias. Attention was given to avoiding double-barreled, lengthy, and negatively worded questions. Additionally, a thorough review of the questionnaire was conducted to guarantee a coherent flow of items, and assessments of face and content validity were performed before seeking expert input.

The questionnaire was self-administered, consisting of four sections. The first section collected sociodemographic data (like gender, age, educational level, weight, and height); the second section asked the participant about knee pain and knee medical history; the third section evaluated awareness regarding osteoarthritis by 25 quotations that were answered by “Yes” or “No” or “ I don't know. The last section evaluated knowledge and precipitation regarding knee pain management. Participants who responded correctly were assigned a score of 1, while those who answered incorrectly or selected "don't know" were assigned a score of 0. The scores were then multiplied by 4, resulting in a scale ranging from 0 to 100. A cutoff point of 60 and above was established to categorize participants as having good knowledge [15].

*Pilot Study*

The questionnaire was already validated. However, in a pilot study, the questionnaire was tested on 10%-15% of the target population to see whether the data collection techniques were correct before beginning a study (data collection tools, reaction of respondents, sampling technique, and proposed work plan). Those who participated in the pilot study were not included in the study population.

*Data Management and Analysis Plan*

The data underwent a thorough review, meticulously scrutinized for completeness, aiming to identify and rectify potential errors, discrepancies, as well as incomplete or inconsistent entries. IBM Corp. Released 2016. IBM SPSS Statistics for Windows, Version 24.0. Armonk, NY: IBM Corp. was used to perform the data analysis. When we entered the data, it was coded first, and then we entered it into the software program. After that, appropriate descriptive statistics were carried out and then summarized as frequency, percentage, and mean. Chi-square tests were used for the analysis of categorical variables. Cronbach's alpha was used to determine the internal consistency of the questionnaire. A value > 0.7 indicates acceptable internal consistency. [16] Finally, the analyzed data was organized and presented in tabular, graphical, and narrative form accordingly. Differences were considered significant at a p-value of <0.05.

*Ethical Consideration* 

Ethics approval was obtained from the ethics committee of Majmaah University (HA-R-088). Informed consent was obtained from the participants. On the first page of the online form, participants who indicated their agreement to take part were subsequently directed to the questionnaire. All collected data was kept confidential and used only for this study.

## Results

The internal validity of the questionnaire was evaluated, and the Cronbach's alpha coefficient was determined to be 0.76, indicating a commendable level of internal consistency. A total of 389 participants completed the questionnaire. More than half (56.6%, n=220) of participants were females, with the majority (66.6%, n=259) aged <50 years and nearly half of them (51.7%, n=201) weighing 60-80 kg; a substantial majority lived in the Riyadh region (27.5%, n=107), with more than half (59.4%, n=231) having a university education and working in offices. 28.3% (n=110); majority (73.3%, n=285) of participants were married, and vast majority (87.9%, n=342) were not smokers. 22.1% (n=86) of the participants reported previous knee injuries, and more than half (52.7%, n=205) were doing average physical activity. 14.1% (n=55) of the respondents reported a previous diagnosis of osteoarthritis, with 35.5% (n=138) of the participants having a family history of osteoarthritis (Table 1).

**Table 1:** Sociodemographic information of the participants (N=389), sociodemographic information category. Sociodemographic information is presented in frequencies (n) and proportions (%)

Sociodemographic information	Category	Frequency and Proportion n (%)
Gender	Male	169 (43.4%)
	Female	220 (56.6%)
Age	<50	259 (66.6%)
	50-60	96 (24.7%)
	60-70	34 (8.7%)
Weight (Kg)	<60	85 (21.9%)
	60-80	201 (51.7%)
	80-100	84 (21.6%)
	100-120	12 (3.1%)
	>120	7 (1.8%)
Height (cm)	<150	31 (8.0%)
	150-160	153 (39.3%)
	160-170	118 (30.3%)
	170-180	77 (19.8%)
	>180	10 (2.6%)
Place of residence	Riyadh region	107 (27.5%)
	Asir region	30 (7.7%)
	Eastern province	105 (27.0%)
	Makkah Region	80 (20.6%)
	Medina region	5 (1.3%)
	Najran region	24 (6.2%)
	Northern border region	3 (0.8%)
	Qassim region	26 (6.7%)
	Tabuk region	3 (0.8%)
	Qunfudhah	1 (0.3%)
	Al Bahah area	1 (0.3%)
	Al-Jawf region	2 (0.5%)
	Doha, Qatar	1 (0.3%)
	Hail region	1 (0.3%)
Education level	Primary	3 (0.8%)
	Secondary	44 (11.3%)
	Diploma	48 (12.3%)
	Middle	6 (1.5%)
	University	231 (59.4%)
	Postgraduate	57 (14.7%)
Occupation	Field work	108 (27.8%)
	Housewife	49 (12.6%)
	Office work	110 (28.3%)
	Retired	70 (18.0%)
	Student	22 (5.7%)
	Unemployed	15 (3.9%)
	Others	15 (3.9%)
Marital status	Single	93 (23.9%)
	Married	285 (73.3%)
	Divorced	5 (1.3%)
	Widow	6 (1.5%)
Smoking status	No	342 (87.9%)
	Yes	37 (9.5%)
	Quit	10 (2.6%)
Previous knee injuries	No	303 (77.9%)
	Yes	86 (22.1%)
Physical activity level	Average activity	205 (52.7%)
	High activity	34 (8.7%)
	Low activity	115 (29.6%)
	Inactive	35 (9.0%)
Previous diagnosis of osteoarthritis	No	333 (85.6%)
	Yes	55 (14.1%)
Family history of osteoarthritis	No	183 (47.0%)
	Yes	138 (35.5%)
	I don't know	68 (17.5%)

In total, 27.8% (n=108) of the respondents had experienced knee joint pain for more than 30 days in the past three months. Of the patients, more than half (51.9%, n=56) had been in pain for less than three months, with the majority (58.3%, n=63) suffering pain in the left knee (40.7%, n=44). According to the findings, 32.4% (n=35) of the respondents noted changes in the size of their knees, with more than half (52.3%, n=64) feeling pain when pressure is placed on their knees, and the majority (80.6%, n=87) reporting pain that comes and goes, with 38.0% (n=41) reporting experiencing knee stiffness, particularly in the first 30 minutes to an hour in the morning (Table 2).

**Table 2:** Knee pain and medical history of the participants Knee pain and medical history of participants are presented in frequencies (n) and proportions (%)

Questions	Categories	Frequency and Proportion n (%)
Have you experienced knee joint pain for more than 30 days in the past 3 months?	Yes	108 (27.8%)
	No	281 (72.2%)
How long have you been in pain?	Less than 3 months	56 (51.9%)
	3 months to 1 year	13 (12.0%)
	1 year to 2 years	5 (4.6%)
	More than 2 years	34 (31.5%)
Do you suffer from knee pain in one or both knees?	One knee	63 (58.3%)
	Two knees	45 (41.7%)
If the pain is in one knee, is it in the right or left knee?	Left	44 (40.7%)
	Right	23 (21.3%)
	Pain in both	41 (38.0%)
Do you notice any change in the size of your knees?	No	73 (67.6%)
	Yes	35 (32.4%)
Do you feel pain when pressure or pressure is placed on your knee(s)?	No	44 (40.7%)
	Yes	64 (52.3%)
How would you describe your knee pain?	Painful	52 (48.1%)
	Very little pain	42 (38.9%)
	Very painful	9 (8.3%)
	unnoticeable	5 (4.6%)
Does your knee pain last throughout the day or does it come and go?	Always constant	21 (19.4%)
	It comes and goes	87 (80.6%)
Do your knees feel stiff during the first 30 minutes to an hour in the morning?	No	67 (62.0%)
	Yes	41 (38.0%)

Of the respondents, 31.1% (n=121) knew how osteoarthritis occurs based on knowledge acquired from the internet (21.1%, n=82) and through education and study (20.1%, n=78). More than half (57.3%, n=223) of the respondents knew someone with osteoarthritis. 88.9% (n=346) of the respondents knew that overweight has a direct relationship with osteoarthritis and that it is a common disease (86.4%, n=336) that can affect different joints (86.6%, n=337). Considering the signs and symptoms of osteoarthritis, 80.2% (n=312) of the participants correctly indicated joint stiffness and swelling (97.9% n=381), and 85.9% (n=334) thought that osteoarthritis can lead to a lack of joint mobility. Regarding the risk factors of osteoarthritis, 79.7% (n=310) of the respondents knew genetic factors; 91.3% (n=355) of them knew age as a risk factor, and more than half (60.7%, n=236) were aware that men and women are affected by osteoarthritis differently. In terms of diagnosis, the majority (84.8%, n=330) correctly cited physical examination and X-rays, while 62.2% (n=242) incorrectly thought a blood test was used for diagnosis. Regarding the treatment, 85.1% (n=331) of the respondents thought that exercise, such as swimming and physical therapy (86.6%, n=337), could improve the symptoms of osteoarthritis, while 92.0% (n=358) believed that joint replacement surgery was ideal for relieving the symptoms of osteoarthritis (Table 3).​​​​​​​

**Table 3:** Awareness of osteoarthritis among the participants Awareness of osteoarthritis among participants is presented in frequencies (n) and proportions (%)

Questions	Categories	Frequency and Proportion n (%)
Do you know how osteoarthritis occurs?	No	268 (68.9%)
	Yes	121 (31.1%)
What is the source of your information?	Education and study	78 (20.1%)
	Internet	82 (21.1%)
	Relative and friends	33 (8.5%)
	There is no source of information	153 (39.3%)
	Communication websites	24 (6.2%)
	Others	19 (4.9%)
Do you know anyone with osteoarthritis?	No	166 (42.7%)
	Yes	223 (57.3%)
Do you think that being overweight has a direct relationship to osteoarthritis?	No	43 (11.1%)
	Yes	346 (88.9%)
Do you think osteoarthritis is rare?	No	336 (86.4%)
	Yes	53 (13.6%)
Do you think it is possible for different joints to be affected by osteoarthritis?	No	52 (13.4%)
	Yes	337 (86.6%)
Do you think osteoarthritis is caused by cold or wet weather?	No	209 (53.7%)
	Yes	180 (46.3%)
Do you think that osteoarthritis is caused by microorganisms?	No	318 (81.7%)
	Yes	71 (18.3%)
Do you think pain is the only symptom of osteoarthritis?	No	248 (63.8%)
	Yes	141 (36.2%)
Do you think joint stiffness is a symptom of osteoarthritis?	No	77 (19.8%)
	Yes	312 (80.2%)
Do you think swelling is a sign of osteoarthritis?	No	8 (2.1%)
	Yes	381(97.9%)
Do you think osteoarthritis can lead to loss of joint mobility?	No	55 (14.1%)
	Yes	334 (85.9%)
Do you think there are genetic factors that can predispose a person to developing osteoarthritis?	No	79 (20.3%)
	Yes	310 (79.7%)
Do you think aging is a risk factor for osteoarthritis?	No	34 (8.7%)
	Yes	355 (91.3%)
Do you think men and women are equally affected by osteoarthritis?	No	236 (60.7%)
	Yes	153 (39.3%)
Do you think a physical examination and x-rays are used to diagnose osteoarthritis?	No	59 (15.2%)
	Yes	330 (84.8%)
Do you think blood tests are used to diagnose osteoarthritis?	No	147 (37.8%)
	Yes	242 (62.2%)
Do you think NSAIDs can improve osteoarthritis symptoms?	No	117 (30.1%)
	Yes	272 (69.9%)
Do you think that some forms of exercise such as swimming are suitable for people with osteoarthritis?	No	58 (14.9%)
	Yes	331 (85.1%)
Do you think acid-free diets are a proven treatment for osteoarthritis?	No	173 (44.5%)
	Yes	216 (55.5%)
Do you think physical therapy can cause significant improvement in osteoarthritis symptoms?	No	52 (13.4%)
	Yes	337 (86.6%)
Do you think that intra-articular injection with stem cells or hyaluronic acid is an effective way to treat arthritis?	No	127 (32.6%)
	Yes	262 (67.4%)
Do you think that intra-articular steroid injections are an effective way to treat arthritis?	No	143 (36.8%)
	Yes	246 (63.2%)
Do you think that joint replacement surgery would be the ideal option to relieve the symptoms of osteoarthritis?	No	31 (8.0%)
	Yes	358 (92.0%)

Table 4 illustrates the knee pain management among the participants. The majority (76.0%, n=82) of the participants had previously been treated for knee pain; 41.8% (n=52) of the participants reported their knee pain being aggravated by climbing stairs, while the pain was relieved by rest (56.4%, n=61) but sometimes they experienced pain at rest (16.7%, n=18). The majority of the participants reported having clicking sounds in the left knee (42.6%, n=46) and in the right knee (39.8%, n=43). According to the results, 35.2% (n=38) of the participants used weight loss to improve their knee pain, with 37.0% (n=40) of the participants reporting painkillers as an effective treatment option. Only 12.0% (n=13) of the respondents reported having an injection of the knee, while the majority (35.2%, n=38) preferred knee exercise. 

**Table 4:** Knee pain management among the participants Knee pain management is presented in frequencies (n) and proportions (%)

Questions	Categories	Frequency and Proportion n (%)
Have you previously been treated for your knee pain, and if yes why?	Yes	82 (76.0%)
	No	26 (24.0%)
What aggravates your knee pain?	Climbing stairs	52 (48.1%)
	Descending stairs	42 (38.9%)
	Kneeling or squatting	9 (8.3%)
	Sitting for long periods of time	5 (4.7%)
What relieves your knee pain?	Rest	61 (56.4%)
	Sitting	25 (23.1%)
	Extending the knee	15 (13.9%)
	Lying down	7 (6.6%)
Do you experience knee pain at rest?	No	76 (70.4%)
	Sometimes	18 (16.7%)
	Mild	8 (7.4%)
	Moderate	4 (3.7%)
	Severe	2 (1.8%)
Other than pain does your right knee have any of the following?	Clicking sound	43 (39.8%)
	Popping sound	36 (33.3%)
	Locking	5 (4.6%)
	Catching	6 (5.6%)
	None	18 (16.7%)
Other than pain does your left knee have any of the following?	Clicking sound	46 (42.6%)
	Popping sound	34 (31.5%)
	Locking	3 (2.8%)
	Catching	4 (3.7%)
	None	21 (19.4%)
How does your knee pain affect your ability to walk?	No difficulty	46 (42.6%)
	Mild difficulty	40 (37.0%)
	Medium difficulty	11 (10.2%)
	Extreme difficulty	5 (4.6%)
	I don’t know	6 (5.6%)
What have you tried to improve your knee pain?	Weight loss	38 (35.2%)
	NSAIDs	35 (32.4%)
	Physical therapy	27 (25.0%)
	Tramadol	5 (4.6%)
	Nothing	3 (2.8%)
Have you ever had an injection in a joint?	Yes	13 (12.0%)
	No	95 (88.0%)
What treatment options improved your quality of life?	Natural therapy	35 (32.4%)
	Painkillers	40 (37.0%)
	Rest	10 (9.3%)
	Exercising/sports	15 (13.9%)
	Nothing	8 (7.4%)
Given the choice of any treatment, which would you prefer and why?	Knee exercise	38 (35.2%)
	Natural therapy	30 (27.8.0%)
	NSAIDs	18 (16.7%)
	Weight lose	15 (13.9%)
	Nothing	7 (6.4%)

Table 5 depicts the relationship between participants’ sociodemographic information and level of knowledge about osteoarthritis. The results established a statistically significant association between age, education level, previous diagnosis of osteoarthritis, family history of osteoarthritis with p-values<0.005 (0.004*, 0.001*, 0.002, and 0.001*), respectively, and the level of knowledge about osteoarthritis. There was no statistically significant association between gender, marital status, smoking status, previous knee injuries, physical activity level, and the level of knowledge about osteoarthritis was (p>0.005). The study results show that 32.9% (n=128) had a good knowledge level, while 67.1% (n=261) had poor knowledge about osteoarthritis.

**Table 5:** The association between sociodemographic information and level of knowledge about osteoarthritis Association between participants’ demographics and knowledge of osteoarthritis. * Significant at the p<0.05 level.

	Level of knowledge			
Variables	Category	Poor	Good	p-value
Gender	Male	112 (66.3%)	57 (33.7%)	0.242
	Female	151 (68.8%)	69 (31.2%)	
Age	<50	169 (65.4%)	90 (34.6%)	0.004*
	50-60	61 (64.0%)	35 (36.0%)	
	60-70	26 (75.1%)	8 (24.9%)	
Educational level	Primary	2 (67.9%)	1 (32.1%)	0.001*
	Secondary	29 (66.8%)	15 (33.2%)	
	Diploma	32 (66.1%)	16 (33.9%)	
	Middle	4 (64.5%)	2 (35.5%)	
	University	147 (63.8%)	84 (36.2%)	
	Postgraduate	36 (63.2%)	21 (36.8%)	
Marital status	Single	62 (66.7%)	31 (33.3%)	0.155
	Married	191 (67.1%)	94 (32.9%)	
	Divorced	3 (68.4%)	2 (31.6%)	
	Widow	4 (66.9%)	2 (33.1%)	
Smoking status	No	232 (67.7%)	110 (32.3%)	0.148
	Yes	24 (66.2%)	13 (33.8%)	
	Quit	7 (68.1%)	3 (31.9%)	
Previous knee injuries	No	203 (66.9%)	100 (33.1%)	0.371
	Yes	58 (67.3%)	28 (32.7%)	
Physical activity level	Average activity	139 (67.8%)	66 (32.2%)	0.537
	High activity	23 (67.2%)	11 (32.8%)	
	Low activity	76 (66.1%)	39 (33.9%)	
	Inactive	24 (69.3%)	11 (30.7%)	
Previous diagnosis of osteoarthritis	No	223 (66.9%)	110 (33.1%)	0.002*
	Yes	37(67.3%)	18 (32.7%)	
Family history of osteoarthritis	No	122 (66.5%)	61 (33.5%)	0.001*
	Yes	93 (67.3%)	45 (32.7%)	
	I don't know	46 (67.9%)	22 (32.1%)	

## Discussion

The study aims to assess the level of awareness regarding osteoarthritis and knee pain management in Saudi Arabia. The sample for the current study primarily consisted of individuals aged less than 50 years, with a predominance of females. The majority of them had a university education, were married, and were doing office work.

The findings revealed that only 32.9% (n = 128) of the participants had good knowledge about osteoarthritis. The finding was consistent with that of the study by Jadhao and Dambhare conducted in China, in which the prevalence of knee osteoarthritis was found to be 34.67% [17]. In the United States, the prevalence rate of knee osteoarthritis was found to range between 30% and 40%, which is similar to the current study [18,19]. The study noted that more than half of the participants had poor knowledge of osteoarthritis, consistent with the studies conducted in Tabuk, Saudi Arabia, by Alqarni et al. [20] and in Jeddah, Saudi Arabia, by Alyami et al. Additionally, the variation in the levels of knowledge across the regions can be attributed to cultural and background differences among the population. In the current study, more than half of the participants were aged less than 50 years.

The study showed that the participants aged 50-60 had better knowledge of osteoarthritis than the ones aged less than 50 years and the ones aged 60-70 years, with male participants showing a higher level of knowledge and awareness about osteoarthritis. The study revealed that individuals who had university and postgraduate levels of education had better knowledge than those with lower levels of education. The findings mirror those of the study conducted by Mukharrib et al., which found that participants whose age was 50 years or older had higher levels of knowledge about osteoarthritis and that those who held a bachelor’s degree had better levels of knowledge about the disease. The increase in knowledge with age can be associated with the fact that osteoarthritis is more prevalent in older age groups than in younger populations.

The study found joint stiffness at 80.2% (n = 312) and swelling at 97.9% (n = 381) to be the most common signs and symptoms of osteoarthritis; the risk factors identified in the study were genetic factors at 79.7% (n = 310) and age at 91.3% (n = 355). The study findings mirror those of the study by Pan, which reported genetic and systematic factors as the major risk factors for osteoarthritis [21]. The treatment options noted by the study were exercise such as swimming (85.1%; n = 331), physical therapy (86.6%; n = 337), and joint replacement surgery (92.0%; n = 358). A similar study conducted by Wei and Dai revealed physical therapy as an effective strategy for the management of knee osteoarthritis [22].

The majority of the participants had previously been treated for knee pain, with most of them reporting that their knee pain was being aggravated by climbing stairs, while in others, the pain was being relieved by rest, although they experienced pain sometimes. The majority of the participants reported having clicking sounds in the left knee (42.6%, n = 46) and in the right knee (39.8%, n = 43). Most of the participants reported using weight loss to improve their knee pain, while others used painkillers as an effective treatment option. The study established a statistically significant association between age, education level, previous diagnosis of osteoarthritis, family history of osteoarthritis with p-values <0.005 (0.004*, 0.001*, 0.002*, and 0.001*), respectively, and the level of knowledge about osteoarthritis. However, there was no statistically significant association between gender, marital status, smoking status, previous knee injuries, physical activity level, and the level of knowledge about osteoarthritis (p > 0.005).

However, it is important to acknowledge several limitations in the methodology employed in this study. First, the use of a cross-sectional design restricts our ability to establish causal relationships between variables. Additionally, the reliance on convenience sampling may affect the generalizability of our findings to a larger population. Moreover, the absence of logistic support necessitated the use of a self-reported method, which introduces the possibility of reporting bias, particularly given the documented tendency for females to report a higher prevalence of OA [23]. Therefore, future research should involve clinical-based diagnoses administered by specialized healthcare providers in order to obtain more robust and accurate results. Additionally, future studies could explore other risk factors that may contribute to the development of knee OA, such as the impact of footwear choices or the knee adduction moment during physical activity. Previous research has suggested that increased knee adduction movement can lead to an exacerbation of knee OA by amplifying the load on the knee's medial side [24]. However, the use of interventions like lateral wedge insoles has been shown to reduce knee adduction movement [25].

## Conclusions

The study revealed that 32.9% (n=128) of the participants had good knowledge about osteoarthritis. The participants aged 50-60 years, those with university and postgraduate levels of education, as well as those who had a previous diagnosis of osteoarthritis and those with a family history of osteoarthritis, had higher and better knowledge and awareness about osteoarthritis. Joint stiffness and swelling were identified as the most common signs and symptoms of osteoarthritis; the risk factors identified in the study were genetic factors and age, while the treatment options noted by the study were exercise, such as swimming, physical therapy, and joint replacement surgery. The study notes the need for enhanced public awareness of the problems associated with osteoarthritis among the Saudi Arabian population.
